# Long-term safety profile of tirabrutinib: final results of a Japanese Phase I study in patients with relapsed or refractory B-cell malignancies

**DOI:** 10.1007/s12185-022-03514-6

**Published:** 2022-12-28

**Authors:** Wataru Munakata, Kiyoshi Ando, Masahiro Yokoyama, Noriko Fukuhara, Kazuhito Yamamoto, Suguru Fukuhara, Ken Ohmachi, Yuko Mishima, Satoshi Ichikawa, Daisuke Ogiya, Arata Aoi, Masahiro Hatsumichi, Kensei Tobinai

**Affiliations:** 1 Department of Hematology, National Cancer Center Hospital, Tokyo, Japan; 2 Department of Hematology and Oncology, Tokai University, Isehara, Japan; 3 Department of Hematology and Oncology, Cancer Institute Hospital, Japanese Foundation for Cancer Research, Tokyo, Japan; 4 Department of Hematology, Tohoku University Graduate School of Medicine, Sendai, Japan; 5 Department of Hematology and Cell Therapy, Aichi Cancer Center, Nagoya, Japan; 6 Department of Clinical Development, Ono Pharmaceutical Co., Ltd., Osaka, Japan

**Keywords:** Tirabrutinib, Long-term safety, B-cell malignancy, B-cell non-Hodgkin lymphoma, Chronic lymphocytic leukemia

## Abstract

Tirabrutinib is a Bruton’s tyrosine kinase inhibitor for treating B-cell malignancies. We report the final results of a Phase I study of tirabrutinib in 17 Japanese patients with B-cell malignancies. Patients were administered tirabrutinib at a dose of 160 mg, 320 mg, or 480 mg once daily, or 300 mg twice daily (*N* = 3, 3, 4, and 7, respectively). Three patients continued tirabrutinib until study completion (November 30, 2020). Adverse events (AEs) occurred in all 17 patients, with Grade 3–4 AEs in 8 (47.1%), serious AEs in 7 (41.2%), drug-related AEs in 16 (94.1%), and Grade 3–4 drug-related AEs in 6 (35.3%). Drug-related AEs reported in 3 or more patients were rash, vomiting, neutropenia, arthralgia, and malaise. One additional serious AE (benign neoplasm of the lung, unrelated to tirabrutinib) occurred after the previous data cutoff (January 4, 2018). Tirabrutinib administration and response assessment were continued for over 4 years in 4 patients. The overall response rate was 76.5% (13/17 patients). The median (range) time to response and duration of response were 0.9 (0.9–5.9) months and 2.59 (0.08–5.45) years, respectively. These findings demonstrate the long-term safety and efficacy of tirabrutinib in Japanese patients with B-cell malignancies.

Clinical trial registration: JapicCTI-142682 (http://www.clinicaltrials.jp/).

**Supplementary Information:**

The online version contains supplementary material available at 10.1007/s12185-022-03514-6.

## Introduction

Bruton’s tyrosine kinase (BTK) is a member of the Tec family of non-receptor protein tyrosine kinases [1, 2] that is highly expressed in hematopoietic cells [3], in which it plays a pivotal role in abnormal B-cell receptor signaling to regulate cell proliferation and survival. Abnormal activity of BTK is observed in a range of B-cell malignancies, including Waldenström macroglobulinemia (WM), chronic lymphocytic leukemia (CLL), and mantle cell lymphoma (MCL) [1, 2]. Therefore, BTK has attracted attention as a potential therapeutic target for these malignancies.

Tirabrutinib (ONO-4059/GS-4059) is a second-generation, highly selective, covalent, irreversible oral inhibitor of BTK that was designed to avoid some limitations of first-generation BTK inhibitors, particularly “off-target” adverse events (AEs) and therapeutic resistance. Preclinical studies provided evidence regarding the cytostatic effects of tirabrutinib in vitro and in vivo [4, 5]. Subsequent clinical trials demonstrated the tolerability and efficacy of tirabrutinib in patients with CLL, MCL, primary central nervous system lymphoma (PCNSL), and WM [6–14], which led to its approval in Japan and South Korea [4, 15, 16]. Tirabrutinib was the first BTK inhibitor to be approved for WM and PCNSL in Japan [15], and for PCNSL in South Korea and Taiwan [16, 17].

In clinical use, like other BTK inhibitors, the administration of tirabrutinib can be continued for as long as it shows clinical efficacy. However, some patients discontinue treatment with BTK inhibitors due to toxicities [18–23]. Second-generation BTK inhibitors, owing to their higher specificity for BTK, may show an improved toxicity profile permitting longer-term administration. However, long-term data are needed to support this possibility. Follow-up data for patients with CLL or MCL were reported in a European Phase I study [9, 11], but further data including its long-term safety profile are limited.

Several Phase I and II clinical studies have been conducted in Japan in patients with relapsed or refractory B-cell malignancies [7], relapsed or refractory PCNSL [8], and WM [10]. In the first of these studies [7], 4 patients were continuing tirabrutinib at the data cutoff (January 4, 2018). Here, we report the long-term safety and efficacy data obtained through to study completion (November 30, 2020).

## Materials and methods

The design of the study (trial registration number: JapicCTI-142682), which adhered to relevant ethical guidelines, including Declaration of Helsinki, was described in more detail in our prior report [7]. The study was approved by the ethics committee/institutional review board at all participating institutions.

Patients with a confirmed histopathological diagnosis and documented history of relapsed or refractory B-cell non-Hodgkin lymphoma [B-NHL: diffuse large B-cell lymphoma (DLBCL), MCL, follicular lymphoma (FL), marginal zone lymphoma, or WM/lymphoplasmacytic lymphoma (LPL)] or CLL/small lymphocytic lymphoma were eligible for this study. The enrollment period was January 2015 to February 2016. All patients provided informed consent. Eligibility criteria are listed in full in the ESM Materials.

Patients were enrolled into 4 dose cohorts (160 mg QD, 320 mg QD, 480 mg QD, and 300 mg BID; cohorts 1–4) in a 3 + 3 dose-escalation scheme. Tirabrutinib was to be continued until progressive disease, unacceptable AEs, or consent withdrawal. Treatment discontinuation, interruption, resumption, and dose modifications were permitted, and the criteria are described in detail in the prior report [7].

Safety was evaluated in terms of AEs and drug-related AEs, laboratory tests, vital signs, and electrocardiography. AEs were assessed using the Common Terminology Criteria for Adverse Events (version 4.0). Drug-related AEs were AEs that were considered to be definitely related, probably related or possibly related to the investigational drug, or AEs with an uncertain relationship to the investigational drug. Five categories of AEs of special interest were also defined as those related to hemorrhage, cytopenia, infection, skin eruption, and diarrhea.

Efficacy was assessed as the best clinical response in accordance with the International Workshop Criteria for NHL, WM, and CLL [24–26]. The overall response rate (ORR) was calculated as the sum of complete responses (CR; CR includes unconfirmed CR or CR with incomplete blood count recovery) and partial responses (PR; PR includes very good PR or PR with residual lymphocytosis). Other efficacy outcomes included the duration of response, event-free survival, progression-free survival (PFS), and overall survival (OS).

The safety analyses were conducted using all enrolled patients who received at least one dose of tirabrutinib (safety analysis set). The efficacy analyses were conducted using all enrolled patients who received at least one dose of tirabrutinib (full analysis set). Data analyses were performed using SAS version 9.4 (SAS Institute, Cary, NC, USA).

## Results

### Patients

As previously described, 17 patients were enrolled and treated in the study, and dose cohorts 1–4 comprised 3, 3, 4, and 7 patients, respectively [7]. There were 5 patients with FL, 4 with non-germinal center B-cell-like (non-GCB) DLBCL, 4 with MCL, 2 with WM, 1 with DLBCL (unknown cell-of-origin), and 1 with CLL (Table 1). The median follow-up through to study completion was 4.3 years, and ranged from 0.04 to 5.8 years. Three patients (MCL: 2 patients; WM: 1 patient) continued tirabrutinib through to study completion (ESM Table 1). One patient with MCL (cohort 3) requested to discontinue tirabrutinib after approximately 2 months of treatment. There were 12 patients with a performance status of 0 and five with a performance status of 1. Approximately half of the patients (9/17, 52.9%) had a history of other medical disorders. Table 1 also lists the frequencies of complications at the time of starting tirabrutinib, including gastrointestinal, cardiovascular, and renal disorders, hypertension, benign prostatic hyperplasia, and abnormal laboratory test values.

**Table 1:** Patient characteristics

*N* (%) of patients	Cohort 1 160 mg QD	Cohort 2 320 mg QD	Cohort 3 480 mg QD	Cohort 4 300 mg BID	Total
	*N* = 3	*N* = 3	*N* = 4	*N* = 7	*N* = 17
Age					
Median, years (range)	70.0 (62–70)	72.0 (68–72)	74.5 (68–79)	71.0 (37–80)	70.0 (37–80)
< 65 years, *n* (%)	1 (33.3)	0	0	1 (14.3)	2 (11.8)
≥ 65 years, *n* (%)	2 (66.7)	3 (100.0)	4 (100.0)	6 (85.7)	15 (88.2)
Sex, *n* (%)					
Male	3 (100.0)	1 (33.3)	1 (25.0)	3 (42.9)	8 (47.1)
Female	0	2 (66.7)	3 (75.0)	4 (57.1)	9 (52.9)
ECOG PS prior to first dose of tirabrutinib, *n* (%)					
0	2 (66.7)	3 (100.0)	2 (50.0)	5 (71.4)	12 (70.6)
1	1 (33.3)	0	2 (50.0)	2 (28.6)	5 (29.4)
Histologic subtype, *n* (%)					
Non-GCB DLBCL	0	0	2 (50.0)	2 (28.6)	4 (23.5)
DLBCL	0	0	0	1 (14.3)	1 (5.9)
MCL	1 (33.3)	2 (66.7)	1 (25.0)	0	4 (23.5)
FL	0	1 (33.3)	1 (25.0)	3 (42.9)	5 (29.4)
WM	1 (33.3)	0	0	1 (14.3)	2 (11.8)
CLL	1 (33.3)	0	0	0	1 (5.9)
Relapsed or refractory after the latest treatment, *n* (%)					
Relapsed	0	2 (66.7)	2 (50.0)	4 (57.1)	8 (47.1)
Refractory	3 (100.0)	1 (33.3)	2 (50.0)	3 (42.9)	9 (52.9)
Prior therapies, *n* (%)					
Prior rituximab	2 (66.7)	3 (100.0)	4 (100.0)	7 (100.0)	16 (94.1)
History of medical disorders, *n* (%)	2 (66.7)	3 (100.0)	1 (25.0)	3 (42.9)	9 (52.9)
Medical complications at the first dose of tirabrutinib, *n* (%)					
Gastrointestinal disorders	2 (66.7)	3 (100.0)	2 (50.0)	3 (42.9)	10 (58.8)
Cardiovascular disorders	0	2 (66.7)	1 (25.0)	1 (14.3)	4 (23.5)
Renal disorders	0	0	2 (50.0)	0	2 (11.8)
Hypertension	2 (66.7)	1 (33.3)	2 (50.0)	3 (42.9)	8 (47.1)
Benign prostatic hyperplasia	1 (33.3)	0	1 (25.0)	1 (14.3)	3 (17.6)
Abnormal laboratory test values	0	0	3 (75.0)	3 (42.9)	6 (35.3)

*BID* twice daily, *CLL* chronic lymphocytic leukemia, *DLBCL* diffuse large B-cell lymphoma, *FL* follicular lymphoma, *MCL* mantle cell lymphoma, *non-GCB DLBCL* non-germinal center B-cell-like diffuse large B-cell lymphoma, *QD* once daily, *WM* Waldenström macroglobulinemia

Modified from Table 1 in [7]

### Safety

AEs occurred in all 17 patients (Table 2), which included Grade 3–4 AEs in 8 patients (47.1%) and serious AEs in 7 (41.2%). AEs led to dose reductions in 3 patients (17.6%) and treatment interruption in 6 patients (35.3%). Tirabrutinib was permanently discontinued in 2 patients due to an AE (acute myeloid leukemia, neuroendocrine carcinoma of the skin), both in patients treated at a dose of 320 mg QD. Acute myeloid leukemia was diagnosed 764 days after starting tirabrutinib; this event was classified as a drug-related AE by the investigator. Sixteen patients (94.1%) experienced a drug-related AE, of which 6 (35.3%) experienced a Grade 3–4 drug-related AE and 4 (23.5%) experienced a serious drug-related AE. Seven patients (41.2%) died during the study, 6 due to cancer progression and 1 due to graft-versus-host disease after undergoing allogeneic transplantation. None of the deaths were related to an AE.

**Table 2:** Summary of adverse events and adverse drug reactions

*N* (%) of patients	Cohort 1 160 mg QD	Cohort 2 320 mg QD	Cohort 3 480 mg QD	Cohort 4 300 mg BID	Total
	*N* = 3	*N* = 3	*N* = 4	*N* = 7	*N* = 17
Any AE	3 (100.0)	3 (100.0)	4 (100.0)	7 (100.0)	17 (100.0)
Any Grade ≥ 3 AE	1 (33.3)	2 (66.7)	2 (50.0)	3 (42.9)	8 (47.1)
AEs leading to death	0 (0.0)	0 (0.0)	0 (0.0)	0 (0.0)	0 (0.0)
Serious AE	1 (33.3)	2 (66.7)	2 (50.0)	2 (28.6)	7 (41.2)
AEs leading to dose reduction^a^	0 (0.0)	1 (33.3)	1 (25.0)	1 (14.3)	3 (17.6)
AEs leading to treatment interruption^a^	2 (66.7)	2 (66.7)	1 (25.0)	1 (14.3)	6 (35.3)
AEs leading to treatment discontinuation^b^	0 (0.0)	2 (66.7)	0 (0.0)	0 (0.0)	2 (11.8)
Any drug-related AE	3 (100.0)	3 (100.0)	4 (100.0)	6 (85.7)	16 (94.1)
Any Grade ≥ 3 drug-related AE	1 (33.3)	2 (66.7)	2 (50.0)	1 (14.3)	6 (35.3)
Drug-related AE leading to death	0 (0.0)	0 (0.0)	0 (0.0)	0 (0.0)	0 (0.0)
Serious drug-related AE	1 (33.3)	1 (33.3)	1 (25.0)	1 (14.3)	4 (23.5)
Drug-related AEs leading to dose reduction^a^	0 (0.0)	1 (33.3)	1 (25.0)	1 (14.3)	3 (17.6)
Drug-related AEs leading to treatment interruption^a^	2 (66.7)	2 (66.7)	1 (25.0)	1 (14.3)	6 (35.3)
Drug-related AEs leading to treatment discontinuation^b^	0 (0.0)	1 (33.3)	0 (0.0)	0 (0.0)	1 (5.9)
Deaths^c^	1 (33.3)	0 (0.0)	2 (50.0)	4 (57.1)	7 (41.2)

*AE* adverse event, *BID* twice daily, *QD* once daily

^a^AEs (drug-related AEs underlined) leading to dose reduction or treatment interruption: neutropenia, Mallory–Weiss syndrome, edema, pyrexia, bronchitis, influenza, hepatitis B reactivation, pneumonia bacterial, herpes ophthalmic, international normalized ratio increased, leukopenia, hyponatremia, hypophosphatemia, pneumonitis, upper respiratory tract inflammation, and rash in one patient each

^b^AEs leading to treatment discontinuation (drug-related AEs underlined): acute myeloid leukemia, neuroendocrine carcinoma of the skin in one patient each

^c^None of the deaths were considered related to AEs

**Fig. 1:**
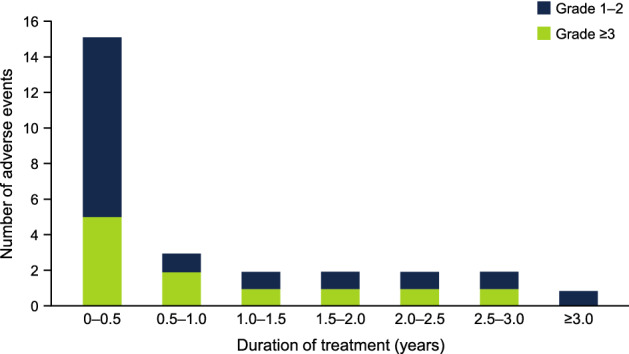
Drug-related adverse events according to the time of onset

Table 3 lists the drug-related AEs that occurred in ≥ 10% of the patients and drug-related Grade 3–4 AEs during the study, and ESM Table 2 lists all of the AEs, in all patients and according to tirabrutinib dose. Drug-related AEs that occurred in 3 or more patients overall were rash [6 patients (35.3%)], vomiting [4 (23.5%)], neutropenia (neutropenia/neutrophil count decreased; 4 (23.5%)], arthralgia [3 (17.6%)], and malaise [3 (17.6%)]. The drug-related Grade 3–4 AEs were neutropenia in 4 patients (23.5%), leukopenia in 2 (11.8%), and anemia, hypophosphatemia, prothrombin time-international normalized ratio (PT-INR) increased, pneumonitis, and acute myeloid leukemia in 1 patient (5.9%) each. As indicated in Fig. 1, most of the patients experienced drug-related AEs within the first ~ 6 months of starting treatment, and one case of acute myeloid leukemia occurred approximately 2 years (diagnosed on day 764) after starting tirabrutinib. No additional drug-related Grade ≥ 3 AEs occurred more than 3 years after starting treatment.

**Table 3:** Drug-related adverse events

*N* (%) of patients	Cohort 1 160 mg QD	Cohort 2 320 mg QD	Cohort 3 480 mg QD	Cohort 4 300 mg BID	Total
	*N* = 3	*N* = 3	*N* = 4	*N* = 7	*N* = 17
Any drug-related AE	3 (100.0)	3 (100.0)	4 (100.0)	6 (85.7)	16 (94.1)
Rash	1 (33.3)	2 (66.7)	2 (50.0)	1 (14.3)	6 (35.3)
Vomiting	0	1 (33.3)	2 (50.0)	1 (14.3)	4 (23.5)
Neutropenia^a^	1 (33.3)	2 (66.7)	1 (25.0)	0	4 (23.5)
Arthralgia	1 (33.3)	1 (33.3)	0	1 (14.3)	3 (17.6)
Malaise	2 (66.7)	0	1 (25.0)	0	3 (17.6)
Nausea	0	0	1 (25.0)	1 (14.3)	2 (11.8)
Diarrhea	0	1 (33.3)	0	1 (14.3)	2 (11.8)
Constipation	0	1 (33.3)	1 (25.0)	0	2 (11.8)
Leukopenia^b^	0	2 (66.7)	0	0	2 (11.8)
Thrombocytopenia^c^	0	1 (33.3)	1 (25.0)	0	2 (11.8)
Anemia	0	0	2 (50.0)	0	2 (11.8)
Hypophosphatemia	0	0	2 (50.0)	0	2 (11.8)
Dysgeusia	0	0	1 (25.0)	1 (14.3)	2 (11.8)
Any drug-related Grade 3–4 AE	1 (33.3)	2 (66.7)	2 (50.0)	1 (14.3)	6 (35.3)
Neutropenia^a^	1 (33.3)	2 (66.7)	1 (25.0)	0	4 (23.5)
Leukopenia^b^	0	2 (66.7)	0	0	2 (11.8)
Anemia	0	0	1 (25.0)	0	1 (5.9)
Hypophosphatemia	0	0	1 (25.0)	0	1 (5.9)
INR increased	0	0	1 (25.0)	0	1 (5.9)
Pneumonitis	0	0	0	1 (14.3)	1 (5.9)
Acute myeloid leukemia	0	1 (33.3)	0	0	1 (5.9)

*AE* adverse event, *BID* twice daily, *INR* international normalized ratio, *QD* once daily

^a^Includes the AE terms neutropenia and neutrophil count decreased

^b^Includes the AE terms leukopenia and white blood cell count decreased

^c^Includes the AE terms thrombocytopenia and platelet count decreased

At the previous cutoff (January 4, 2018), 9 serious AEs had been reported in 7 patients (4 were considered drug related) [7]. In the time between the earlier cutoff date and study completion, one additional serious AE (benign neoplasm of the lung) was reported at 1681 days after starting treatment in a patient treated with 300 mg BID. This serious AE was not considered related to tirabrutinib and the patient recovered.

Five AE categories of special interest were defined (Table 4), with AEs related to hemorrhage, cytopenia, infection, skin eruption, and diarrhea in 4 (23.5%), 5 (29.4%), 8 (47.1%), 10 (58.8%), and 4 (23.5%) patients, respectively. Although not defined as AEs of special interest, there were no cases of atrial fibrillation or hypertension.

**Table 4:** Frequencies of adverse events of specific interest

*N* (%) of patients	Cohort 1 160 mg QD	Cohort 2 320 mg QD	Cohort 3 480 mg QD	Cohort 4 300 mg BID	Total
	*N* = 3	*N* = 3	*N* = 4	*N* = 7	*N* = 17
Any AE of special interest	3 (100.0)	3 (100.0)	3 (75.0)	6 (85.7)	15 (88.2)
AEs related to hemorrhage	1 (33.3)	0	1 (25.0)	2 (28.6)	4 (23.5)
Ear hemorrhage	1 (33.3)	0	0	0	1 (5.9)
INR increased	0	0	1 (25.0)	0	1 (5.9)
Mallory–Weiss syndrome	0	0	1 (25.0)	0	1 (5.9)
Hematuria	0	0	0	1 (14.3)	1 (5.9)
Purpura	0	0	0	1 (14.3)	1 (5.9)
AEs related to cytopenia	1 (33.3)	1 (33.3)	2 (50.0)	1 (14.3)	5 (29.4)
Neutropenia^a^	1 (33.3)	2 (66.7)	1 (25.0)	0	4 (23.5)
Anemia	0	0	2 (50.0)	1 (14.3)	3 (17.6)
Leukopenia^b^	0	2 (66.7)	0	0	2 (11.8)
Thrombocytopenia^c^	0	1 (33.3)	1 (25.0)	0	2 (11.8)
Febrile neutropenia	1 (33.3)	0	0	0	1 (5.9)
Iron deficiency anemia	1 (33.3)	0	0	0	1 (5.9)
AEs related to infection	3 (100.0)	1 (33.3)	0	4 (57.1)	8 (47.1)
Nasopharyngitis	1 (33.3)	0	0	1 (14.3)	2 (11.8)
Pharyngitis	1 (33.3)	0	0	1 (14.3)	2 (11.8)
Sinusitis	1 (33.3)	0	0	1 (14.3)	2 (11.8)
Cystitis	0	1 (33.3)	0	1 (14.3)	2 (11.8)
Conjunctivitis viral	1 (33.3)	0	0	0	1 (5.9)
Eye infection	1 (33.3)	0	0	0	1 (5.9)
Folliculitis	1 (33.3)	0	0	0	1 (5.9)
Infection	1 (33.3)	0	0	0	1 (5.9)
Herpes ophthalmic	1 (33.3)	0	0	0	1 (5.9)
Anal abscess	1 (33.3)	0	0	0	1 (5.9)
Hepatitis B reactivation	1 (33.3)	0	0	0	1 (5.9)
Acarodermatitis	0	1 (33.3)	0	0	1 (5.9)
Influenza	0	0	0	1 (14.3)	1 (5.9)
Periodontitis	0	0	0	1 (14.3)	1 (5.9)
Pneumonia bacterial	0	0	0	1 (14.3)	1 (5.9)
Oral herpes	0	0	0	1 (14.3)	1 (5.9)
Bronchitis	0	0	0	1 (14.3)	1 (5.9)
AEs related to skin eruption	3 (100.0)	3 (100.0)	2 (50.0)	2 (28.6)	10 (58.8)
Rash	1 (33.3)	2 (66.7)	2 (50.0)	1 (14.3)	6 (35.3)
Prurigo	1 (33.3)	0	0	0	1 (5.9)
Seborrheic dermatitis	1 (33.3)	0	0	0	1 (5.9)
Skin disorder	0	1 (33.3)	0	0	1 (5.9)
Toxic skin eruption	0	0	0	1 (14.3)	1 (5.9)
AEs related to diarrhea	1 (33.3)	1 (33.3)	0	2 (28.6)	4 (23.5)
Diarrhea	0	1 (33.3)	0	2 (28.6)	3 (17.6)
Colitis ischemic	1 (33.3)	0	0	0	1 (5.9)

*AE* adverse event, *BID* twice daily, *INR* international normalized ratio, *QD* once daily

^a^Includes the AE terms neutropenia and neutrophil count decreased

^b^Includes the AE terms leukopenia and white blood cell count decreased

^c^Includes the AE terms thrombocytopenia and platelet count decreased

### Treatment exposure and response

**Fig. 2:**
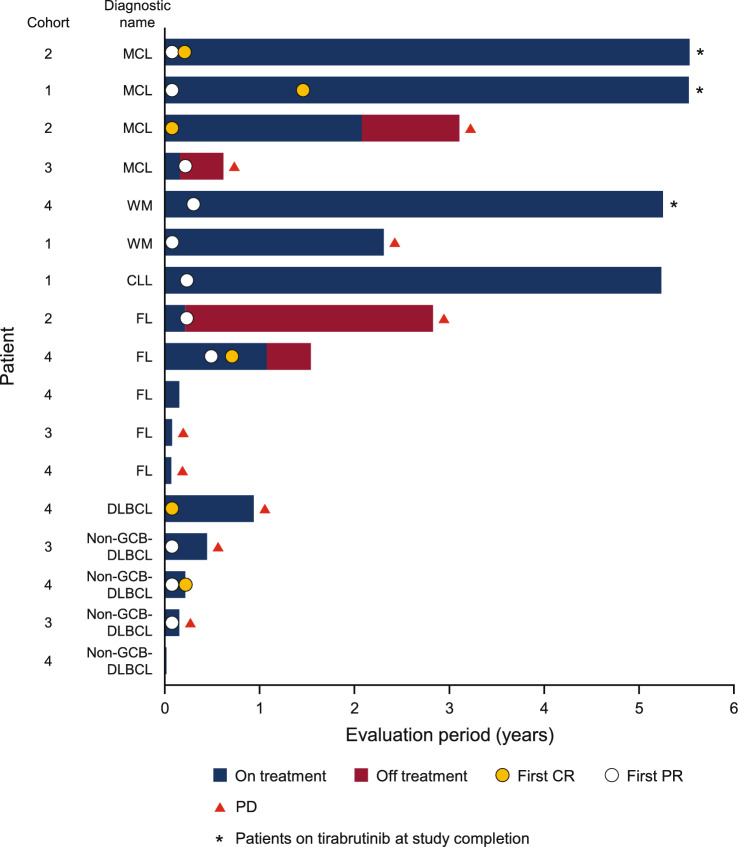
Treatment exposure and duration of response: “On treatment” indicates patients were receiving tirabrutinib. “Off treatment” indicates patients had stopped administration of tirabrutinib but were being followed up in the study. Cohort 1: 160 mg QD; Cohort 2: 320 mg QD; Cohort 3: 480 mg QD; Cohort 4: 300 mg BID. *BID* twice daily, *CLL* chronic lymphocytic leukemia, *CR* complete response, *DLBCL* diffuse large B-cell lymphoma, *FL* follicular lymphoma, *MCL* mantle cell lymphoma, *non-GCB DLBCL* non-germinal center B-cell-like diffuse large B-cell lymphoma, *PD* progressive disease, *PR* partial response, *QD* once daily, *WM* Waldenström macroglobulinemia

Figure 2 shows the swimmers plot for treatment exposure and response in individual patients according to the type of malignancy and the dose of tirabrutinib. The administration of tirabrutinib and a response to treatment were continued for over 4 years in 2 patients with MCL, 1 with WM, and 1 with CLL. The ORR was 76.5% (13/17 patients), with a median time to response of 0.9 months (range 0.9–5.9 months) and the median duration of response was 2.59 years (range 0.08–5.45 years). The ORR according to the type of malignancy is summarized in ESM Table 3; values are unchanged from the previous report [7]. The median PFS was 2.82 years (95% confidence interval 0.44 years to not reached) and median OS was not reached. The PFS and OS values according to the type of malignancy are summarized in ESM Table 3.

## Discussion

This Phase I study was performed to investigate the maximum tolerated dose of tirabrutinib, as well as its safety, efficacy, and pharmacokinetics, in Japanese patients with relapsed or refractory B-cell NHL or CLL. Results at an earlier cutoff (January 4, 2018) were reported in our previous manuscript [7]. Here, we extend these findings with a longer follow-up of patients, except for 1 patient with MCL in cohort 3 who discontinued tirabrutinib approximately 2 months after starting treatment. Four patients were treated with tirabrutinib for > 5 years, of which 3 continued treatment through to study completion (November 30, 2020). From a safety perspective, the most important findings of the current paper include the frequencies of drug-related AEs and AEs of special interest. Drug-related AEs that occurred in 3 or more patients overall were rash, vomiting, neutropenia, arthralgia, and malaise, and drug-related Grade 3–4 AEs were neutropenia, leukopenia, anemia, hypophosphatemia, PT-INR increased, pneumonitis, and acute myeloid leukemia. It is notable that the majority of drug-related AEs (any Grade, Grade 3–4) occurred within the first 6 months of treatment, and no drug-related Grade 3–4 AEs occurred > 3 years after starting treatment. By comparison, Grade ≥ 3 AEs of clinical interest were reported more than 3 years after starting ibrutinib [27]. In our study, AEs related to hemorrhage, cytopenia, infection, skin eruption, and diarrhea occurred in 4 (23.5%), 5 (29.4%), 8 (47.1%), 10 (58.8%), and 4 (23.5%) patients. From an efficacy perspective, the ORR was unchanged (76.5%) and the median duration of response was extended to 2.59 years (range 0.08–5.45 years) with the longer follow-up of patients.

The safety data in this study are generally consistent with those in prior studies of tirabrutinib. For example, in prior Japanese studies, rash, hematologic AEs, erythema multiforme, and constipation were frequent AEs in a Phase I/II study of 44 patients with PCNSL [8], and rash, hematologic AEs, and stomatitis were the most common AEs in a Phase II study of 27 patients with WM [10]. In international studies, other frequently reported AEs included hematologic AEs, diarrhea, petechiae, rash, nasopharyngitis, upper respiratory tract infection, fatigue, cough, and arthralgia [12, 13].

The Japanese product label for ibrutinib, a first-generation BTK inhibitor, lists 11 categories of adverse drug reactions (ADR) as identified/potential risks in the risk management plan (hemorrhage, bone marrow suppression, infection, arrhythmia, hypersensitivity, tumor lysis syndrome, liver failure/dysfunction, interstitial lung disease, secondary malignant tumors, leukostasis, and oculomucocutaneous syndrome) [5]. It is notable that some of these types of events, including leukostasis, progressive multifocal leukoencephalopathy, arrhythmia, tumor lysis syndrome, hypersensitivity, and oculomucocutaneous syndrome, did not occur in the present study. Atrial fibrillation has been acknowledged as a common reason for discontinuing ibrutinib [20, 28] and concern has been raised regarding hypertension in patients treated with ibrutinib [29–31]. Although cardiovascular disorders and hypertension were present in 4 (23.5%) and 8 (47.1%) of patients prior to starting tirabrutinib, no new cases of atrial fibrillation or hypertension were reported in our study.

Hemorrhage-related AEs are a potential class effect of BTK inhibitors. In our study, these events included ear hemorrhage, hematuria, PT-INR increased, Mallory–Weiss syndrome, and purpura, each of which occurred in one patient (5.9%) (Table 4). Of these, PT-INR increased was Grade ≥ 3 and Mallory–Weiss syndrome was classified as a serious AE. Cerebral hemorrhage and gastrointestinal hemorrhage, which are listed as ADRs associated with ibrutinib [5], were not observed in our study. Because hemorrhage-related AEs were observed in this study and some were Grade ≥ 3 or serious, special attention should be paid to the occurrence of such AEs in the future clinical studies of tirabrutinib and in clinical practice.

Infections were also evaluated as AEs of specific interest, and included cystitis, nasopharyngitis, pharyngitis, and sinusitis in 2 patients each (11.8%) (Table 4). There was one Grade ≥ 3 infection-related AE (pneumonia bacterial). All of the infection-related AEs were classified as recovered, with the exception of one case of sinusitis. None of the AEs required treatment interruption and none were considered related to tirabrutinib, except for one case of cystitis. Although the infection-related AEs were manageable in most patients with prophylactic use or treatment with antibacterial agents, some caution may be necessary, especially in patients with serious infections.

Myelosuppression-related AEs included neutropenia/neutrophil count decreased in 4 patients (23.5%), anemia in 3 patients (17.6%), thrombocytopenia/platelet count decreased in 2 patients (11.8%), and leukopenia/white blood cell count decreased in 2 patients (11.8%). These values are generally similar to those reported in the package insert for ibrutinib (anemia, 9.6%; neutropenia, 12.5%; thrombocytopenia, 9.8%) [5].

Rash, diarrhea, headache, nausea, fatigue, and myalgia have been reported for other second-generation BTK inhibitors [32–34]. In earlier studies, rash, diarrhea, headache, nausea, fatigue, and myalgia were reported in up to 33%, 58%, 69%, 27%, 21%, and 38% of patients in clinical trials of acalabrutinib [35, 36]. Rash and diarrhea were reported in up to 34% and up to 21% of patients in clinical trials of zanubrutinib [22, 37]. In other studies of tirabrutinib, rash and diarrhea both occurred in up to 44% of the patients [9, 10]. In the present study, rash, diarrhea, headache, nausea, fatigue, and myalgia occurred in 35.3%, 17.6%, 11.8%, 17.6%, 0%, and 5.9% of the patients, respectively. Based on these data, there is a need to pay special attention to these types of AEs in patients treated with tirabrutinib, as with other BTK inhibitors.

From an efficacy perspective, although this was a Phase I study with a limited number of patients, the study enrolled several patients with MCL, WM, and CLL who were treated with tirabrutinib for a long period of time with long responses. These data suggest that tirabrutinib may be effective in patients with MCL, WM, and CLL.

Limitations of this Phase I study include its small sample, dose-escalation design, and the inclusion of multiple subtypes of B-NHL and CLL. These factors may limit the generalizability of the results to broader clinical practice. However, these limitations must be considered in relation to the primary objective of this Phase I study, which was to evaluate the safety and detect potential dose-limiting toxicities, and hence the recommended dose in Japanese patients. Larger studies may be necessary to confirm our results.

With a median follow-up of 4.3 years, this study comprises the longest data set of any tirabrutinib study published to date and 4 patients were continuing treatment for ≥ 5 years. This allowed us to evaluate the long-term safety of tirabrutinib. We observed no additional risks or emergence of Grade ≥ 3 drug-related AEs since the prior cutoff at which time the median follow-up was 2.3 years. The current results, as well as those from the prior report, demonstrate the long-term tolerability of tirabrutinib at doses of 480 mg QD or 300 mg BID in patients with relapsed or refractory B-NHL or CLL, with a low rate of discontinuation due to AEs/drug-related AEs.

## Supplementary Information

Below is the link to the electronic supplementary material.

Supplementary file1 (DOC 231 KB)

## Data Availability

Qualified researchers may request Ono Pharma to disclose individual patient-level data from clinical studies through the following website: https://www.clinicalstudydatarequest.com/. For more information on Ono Pharma’s Policy for the Disclosure of Clinical Study Data, please see the following website: https://www.ono.co.jp/eng/rd/policy.html.
