# Supplementary material for: Long-term safety profile of tirabrutinib: final results of a Japanese Phase I study in patients with relapsed or refractory B-cell malignancies
Source: Int J Hematol. 2022 Dec 28;117(4):553–62. doi: 10.1007/s12185-022-03514-6 (PMC10063512; doi:10.1007/s12185-022-03514-6)
Supplement: Supplementary file 1 — Supplementary file1 (DOC 231 KB) [file 12185_2022_3514_MOESM1_ESM.doc]

# Electronic Supplementary Material

**Long-term safety profile of tirabrutinib: final results of a Japanese Phase I study in patients with relapsed or refractory B-cell malignancies**

**Contents Page**

Inclusion criteria 2

Exclusion criteria 3

ESM Table 1 6

ESM Table 2 7

ESM Table 3 13

***Inclusion criteria***

Patients who satisfied all of the following inclusion criteria were eligible for the study:

1. Provision of written informed consent.
2. Inpatient (regardless of sex) until completion of assessments on Cycle 1, Day 15.
3. Age ≥20 years at the time of providing consent.
4. Confirmed diagnosis of and documented history of relapsed or refractory B-NHL/CLL for which no therapy of curative or high priority exists and for whom treatment with a BTK inhibitor may be deemed appropriate. Eligible B-NHL patients include relapsed or refractory diffuse large B-cell lymphoma, mantle cell lymphoma, indolent lymphoma (including but not limited to follicular lymphoma [FL], small lymphocytic lymphoma, marginal zone lymphoma [MZL] or Waldenström’s macroglobulinemia [WM]).
   Definition of relapsed/refractory:
5. Relapsed: patients who achieved CR, CRu, CRi or PR with a duration of ≥6 months after the last treatment.
6. Refractory: patients who did not achieve CR, CRu, CRi or PR after last treatment or achieved CR, CRu, CRi or PR with a duration of <6 months after the last treatment.
7. ≥1 previous treatments for B-NHL/CLL.
8. Patients with a bi-dimensionally measurable lesion >1.5 cm (largest dimension) on computed tomography (CT). For CLL patients with a lymphocyte count of ≥5,000/μL and WM patients, only those without a lymphoma lesion can be registered.
9. Eastern Cooperative Oncology Group performance status of ≤2.
10. Life expectancy ≥90 days.
11. Females of childbearing potential (including females without menses for medical reasons such as chemotherapy-induced amenorrhea) must agree to use an effective method of dual contraception during the study and for 120 days following the last dose of the study drug.
12. Male patients who agree to use dual contraception from the start of study treatment to ≥180 days after the last dose of the study drug.
13. Patients with CLL if they meet criteria 1–10 plus at least one of the following criteria:
    1. Rai Stage III/IV or Binet Stage C disease
    2. Rai Stage I/II or Binet Stage B disease requiring treatment.

***Exclusion criteria***

Patients who met any of the following criteria were excluded from the study:

1. CLL patients with uncontrolled autoimmune hemolytic anemia (AIHA) or with a medical history/current Richter’s syndrome.
2. Current/history of pancreatitis.
3. Current/history of parkinsonian syndrome, cerebellar disorders, or other movement-related disorder.
4. Central nervous system (CNS) lymphoma.
5. Inability to swallow capsules or history of malabsorption/malabsorption syndrome, disease significantly affecting gastrointestinal function, complete resection of the stomach or small bowel, ulcerative colitis, symptomatic inflammatory bowel disease, partial or complete bowel obstruction.
6. History of severe allergic or anaphylactic reactions.
7. Prior use of standard anti-lymphoma/leukemia therapy or radiation therapy within 28 days before the first dose of the study drug.
8. Prior administration of radio-immunotherapy within 90 days before the first dose of the study drug.
9. Major surgery (other than for diagnostic purposes) within 28 days or minor surgery within 1 week before the first dose of the study drug.
10. Prior treatment with the study drug.
11. Patients who received any other unapproved drug (including an unapproved combination or new formulation drug in clinical research) within 28 days before the first dose of the study drug.
12. Ongoing systemic corticosteroid use, exception for corticosteroids to control symptoms related to underlying disease and/or corticosteroid use for other indications, with a maximum permitted dose of 20 mg/day prednisone or equivalent.
13. History of another malignancy, which could affect compliance with the protocol or interpretation of results.
14. History of malignancy other than B-NHL/CLL that has been treated, but not with a curative intent, unless the malignancy has been in remission without treatment for ≥2 years prior to enrollment.
15. Evidence of significant, uncontrolled concomitant diseases, which could affect compliance with the protocol or interpretation of results, including significant cardiovascular disease (such as New York Heart Association Class III or IV cardiac disease, myocardial infarction within the last 6 months, unstable arrhythmias, or unstable angina) or pulmonary disease (including obstructive pulmonary disease and history of symptomatic bronchospasm).
16. Known active bacterial, viral (including human immunodeficiency virus [HIV]), fungal, mycobacterial, or other infection (excluding fungal infections of nail beds) or any major episode of infection requiring hospitalization or treatment with IV antibiotics within 28 days before receiving the first dose of the study drug.
17. Any of the following abnormal laboratory values at screening:

a. Creatinine clearance <50 mL/min (calculated using Cockcroft-Gault formula) or 50 mL/min/1.73 m^2^ (Modification of Diet in Renal Disease [MDRD] formula). However, patients with estimated creatinine clearance under 50 mL/min could be eligible if the measured creatinine clearance (based on 24-hour urine collection or other reliable method) was ≥50 mL/min.

b. Aspartate aminotransferase (AST) or alanine aminotransferase (ALT) ≥2.5 times the upper limit of normal

c. Platelet count <50 × 10^9^/L

d. Neutrophils <1.0 × 10^9^/L

e. Hemoglobin <8.0 g/dL

f. Total bilirubin ≥1.5 times the upper limit of normal.

1. Patients with a positive result for any of the following tests: HIV-1 antibody or HIV-2 antibody test, HTLV-1 antibody test, HBs antigen test, and HCV antibody test. Patients who are HBs antigen negative are to be excluded if they are positive for HBs antibody or HBc antibody and HBV-DNA has been detected by quantitative analysis.
2. Women who are pregnant or lactating. Lactating women could not be registered after discontinuation of breastfeeding.
3. Patients unable to provide informed consent due to dementia or other cognitive impairment.
4. Patients considered unsuitable for enrollment in the study in the opinion of the principal investigator or sub-investigator.

**ESM Table 1** Treatment disposition

| ***N* (%) of patients** | **Cohort 1 160 mg QD** | **Cohort 2 320 mg QD** | **Cohort 3 480 mg QD** | **Cohort 4 300 mg BID** | **Total** |
| --- | --- | --- | --- | --- | --- |
|  | ***N* = 3** | ***N* = 3** | ***N* = 4** | ***N* = 7** | ***N* = 17** |
| Continuation of treatment through to study completion | 1 (33.3) | 1 (33.3) | 0 | 1 (14.3) | 3 (17.6) |
| Discontinuation of treatment | 2 (66.7) | 2 (66.7) | 4 (100.0) | 6 (85.7) | 14 (82.4) |
| Progressive disease | 1 (33.3) | 0 | 3 (75.0) | 2 (28.6) | 6 (35.3) |
| The investigator/sub-investigator considered it difficult to continue due to AEs | 0 | 2 (66.7) | 0 | 0 | 2 (11.8) |
| The investigator/sub-investigator considered that study continuation was inappropriate | 1 (33.3) | 0 | 0 | 4 (57.1) | 5 (29.4) |

*AE* adverse event, *BID* twice daily, *QD* once daily

**ESM Table 2** Adverse events

| ***N* (%) of patients** | **Cohort 1 160 mg QD** | **Cohort 2 320 mg QD** | **Cohort 3 480 mg QD** | **Cohort 4 300 mg BID** | **Total** |
| --- | --- | --- | --- | --- | --- |
|  | ***N* = 3** | ***N* = 3** | ***N* = 4** | ***N* = 7** | ***N* = 17** |
| Total | 3 (100.0) | 3 (100.0) | 4 (100.0) | 7 (100.0) | 17 (100.0) |
| **AEs by preferred term** |  |  |  |  |  |
| Rash | 1 (33.3) | 2 (66.7) | 2 (50.0) | 1 (14.3) | 6 (35.3) |
| Vomiting | 0 | 1 (33.3) | 2 (50.0) | 2 (28.6) | 5 (29.4) |
| Arthralgia | 2 (66.7) | 1 (33.3) | 0 | 1 (14.3) | 4 (23.5) |
| Neutropenia ^a^ | 1 (33.3) | 2 (66.7) | 1 (25.0) | 0 | 4 (23.5) |
| Upper respiratory tract inflammation | 2 (66.7) | 1 (33.3) | 0 | 0 | 3 (17.6) |
| Malaise | 2 (66.7) | 0 | 1 (25.0) | 0 | 3 (17.6) |
| Constipation | 0 | 1 (33.3) | 1 (25.0) | 1 (14.3) | 3 (17.6) |
| Diarrhea | 0 | 1 (33.3) | 0 | 2 (28.6) | 3 (17.6) |
| Anemia | 0 | 0 | 2 (50.0) | 1 (14.3) | 3 (17.6) |
| Nausea | 0 | 0 | 1 (25.0) | 2 (28.6) | 3 (17.6) |
| Insomnia | 0 | 0 | 0 | 3 (42.9) | 3 (17.6) |
| Nasopharyngitis | 1 (33.3) | 0 | 0 | 1 (14.3) | 2 (11.8) |
| Pharyngitis | 1 (33.3) | 0 | 0 | 1 (14.3) | 2 (11.8) |
| Sinusitis | 1 (33.3) | 0 | 0 | 1 (14.3) | 2 (11.8) |
| Back pain | 1 (33.3) | 0 | 0 | 1 (14.3) | 2 (11.8) |
| Headache | 1 (33.3) | 0 | 0 | 1 (14.3) | 2 (11.8) |
| Leukopenia ^b^ | 0 | 2 (66.7) | 0 | 0 | 2 (11.8) |
| Lymphocyte count decreased | 0 | 1 (33.3) | 1 (25.0) | 0 | 2 (11.8) |
| Thrombocytopenia ^c^ | 0 | 1 (33.3) | 1 (25.0) | 0 | 2 (11.8) |
| Cystitis | 0 | 1 (33.3) | 0 | 1 (14.3) | 2 (11.8) |
| Gamma-glutamyltransferase increased | 0 | 1 (33.3) | 0 | 1 (14.3) | 2 (11.8) |
| Hypokalemia | 0 | 0 | 2 (50.0) | 0 | 2 (11.8) |
| Hypophosphatemia | 0 | 0 | 2 (50.0) | 0 | 2 (11.8) |
| Aspartate aminotransferase increased | 0 | 0 | 1 (25.0) | 1 (14.3) | 2 (11.8) |
| Dysgeusia | 0 | 0 | 1 (25.0) | 1 (14.3) | 2 (11.8) |
| Pyrexia | 0 | 0 | 0 | 2 (28.6) | 2 (11.8) |
| Febrile neutropenia | 1 (33.3) | 0 | 0 | 0 | 1 (5.9) |
| Iron deficiency anemia | 1 (33.3) | 0 | 0 | 0 | 1 (5.9) |
| Deafness | 1 (33.3) | 0 | 0 | 0 | 1 (5.9) |
| Ear hemorrhage | 1 (33.3) | 0 | 0 | 0 | 1 (5.9) |
| Vertigo positional | 1 (33.3) | 0 | 0 | 0 | 1 (5.9) |
| Blepharitis | 1 (33.3) | 0 | 0 | 0 | 1 (5.9) |
| Abdominal pain upper | 1 (33.3) | 0 | 0 | 0 | 1 (5.9) |
| Anal fistula | 1 (33.3) | 0 | 0 | 0 | 1 (5.9) |
| Colitis ischemic | 1 (33.3) | 0 | 0 | 0 | 1 (5.9) |
| Gastritis | 1 (33.3) | 0 | 0 | 0 | 1 (5.9) |
| Conjunctivitis viral | 1 (33.3) | 0 | 0 | 0 | 1 (5.9) |
| Eye infection | 1 (33.3) | 0 | 0 | 0 | 1 (5.9) |
| Folliculitis | 1 (33.3) | 0 | 0 | 0 | 1 (5.9) |
| Infection | 1 (33.3) | 0 | 0 | 0 | 1 (5.9) |
| Anal abscess | 1 (33.3) | 0 | 0 | 0 | 1 (5.9) |
| Hepatitis B reactivation | 1 (33.3) | 0 | 0 | 0 | 1 (5.9) |
| Herpes ophthalmic | 1 (33.3) | 0 | 0 | 0 | 1 (5.9) |
| Rib fracture | 1 (33.3) | 0 | 0 | 0 | 1 (5.9) |
| Myalgia | 1 (33.3) | 0 | 0 | 0 | 1 (5.9) |
| Dizziness | 1 (33.3) | 0 | 0 | 0 | 1 (5.9) |
| Oropharyngeal pain | 1 (33.3) | 0 | 0 | 0 | 1 (5.9) |
| Dermal cyst | 1 (33.3) | 0 | 0 | 0 | 1 (5.9) |
| Dermatitis | 1 (33.3) | 0 | 0 | 0 | 1 (5.9) |
| Eczema | 1 (33.3) | 0 | 0 | 0 | 1 (5.9) |
| Miliaria | 1 (33.3) | 0 | 0 | 0 | 1 (5.9) |
| Prurigo | 1 (33.3) | 0 | 0 | 0 | 1 (5.9) |
| Seborrheic dermatitis | 1 (33.3) | 0 | 0 | 0 | 1 (5.9) |
| Eye discharge | 0 | 1 (33.3) | 0 | 0 | 1 (5.9) |
| Edema | 0 | 1 (33.3) | 0 | 0 | 1 (5.9) |
| Acarodermatitis | 0 | 1 (33.3) | 0 | 0 | 1 (5.9) |
| Blood triglycerides increased | 0 | 1 (33.3) | 0 | 0 | 1 (5.9) |
| C-reactive protein increased | 0 | 1 (33.3) | 0 | 0 | 1 (5.9) |
| Neck pain | 0 | 1 (33.3) | 0 | 0 | 1 (5.9) |
| Acute myeloid leukemia | 0 | 1 (33.3) | 0 | 0 | 1 (5.9) |
| Neuroendocrine carcinoma of the skin | 0 | 1 (33.3) | 0 | 0 | 1 (5.9) |
| Neuropathy peripheral | 0 | 1 (33.3) | 0 | 0 | 1 (5.9) |
| Skin disorder | 0 | 1 (33.3) | 0 | 0 | 1 (5.9) |
| Dry mouth | 0 | 0 | 1 (25.0) | 0 | 1 (5.9) |
| Mallory–Weiss syndrome | 0 | 0 | 1 (25.0) | 0 | 1 (5.9) |
| Stomatitis | 0 | 0 | 1 (25.0) | 0 | 1 (5.9) |
| Spinal compression fracture | 0 | 0 | 1 (25.0) | 0 | 1 (5.9) |
| Blood lactate dehydrogenase increased | 0 | 0 | 1 (25.0) | 0 | 1 (5.9) |
| INR increased | 0 | 0 | 1 (25.0) | 0 | 1 (5.9) |
| Hyponatremia | 0 | 0 | 1 (25.0) | 0 | 1 (5.9) |
| Decreased appetite | 0 | 0 | 1 (25.0) | 0 | 1 (5.9) |
| Arthritis | 0 | 0 | 1 (25.0) | 0 | 1 (5.9) |
| Spinal column stenosis | 0 | 0 | 1 (25.0) | 0 | 1 (5.9) |
| Restless legs syndrome | 0 | 0 | 1 (25.0) | 0 | 1 (5.9) |
| Cough | 0 | 0 | 1 (25.0) | 0 | 1 (5.9) |
| Skin exfoliation | 0 | 0 | 1 (25.0) | 0 | 1 (5.9) |
| Vertigo | 0 | 0 | 0 | 1 (14.3) | 1 (5.9) |
| Abdominal pain | 0 | 0 | 0 | 1 (14.3) | 1 (5.9) |
| Large intestine polyp | 0 | 0 | 0 | 1 (14.3) | 1 (5.9) |
| Epigastric discomfort | 0 | 0 | 0 | 1 (14.3) | 1 (5.9) |
| Bronchitis | 0 | 0 | 0 | 1 (14.3) | 1 (5.9) |
| Influenza | 0 | 0 | 0 | 1 (14.3) | 1 (5.9) |
| Periodontitis | 0 | 0 | 0 | 1 (14.3) | 1 (5.9) |
| Pneumonia bacterial | 0 | 0 | 0 | 1 (14.3) | 1 (5.9) |
| Oral herpes | 0 | 0 | 0 | 1 (14.3) | 1 (5.9) |
| Excoriation | 0 | 0 | 0 | 1 (14.3) | 1 (5.9) |
| Procedural pain | 0 | 0 | 0 | 1 (14.3) | 1 (5.9) |
| Alanine aminotransferase increased | 0 | 0 | 0 | 1 (14.3) | 1 (5.9) |
| Blood creatinine increased | 0 | 0 | 0 | 1 (14.3) | 1 (5.9) |
| Blood alkaline phosphatase increased | 0 | 0 | 0 | 1 (14.3) | 1 (5.9) |
| Hyperglycemia | 0 | 0 | 0 | 1 (14.3) | 1 (5.9) |
| Hyperlipidemia | 0 | 0 | 0 | 1 (14.3) | 1 (5.9) |
| Benign lung neoplasm | 0 | 0 | 0 | 1 (14.3) | 1 (5.9) |
| Hypoesthesia | 0 | 0 | 0 | 1 (14.3) | 1 (5.9) |
| Anxiety | 0 | 0 | 0 | 1 (14.3) | 1 (5.9) |
| Hematuria | 0 | 0 | 0 | 1 (14.3) | 1 (5.9) |
| Urinary retention | 0 | 0 | 0 | 1 (14.3) | 1 (5.9) |
| Renal impairment | 0 | 0 | 0 | 1 (14.3) | 1 (5.9) |
| Dysphonia | 0 | 0 | 0 | 1 (14.3) | 1 (5.9) |
| Hypoxia | 0 | 0 | 0 | 1 (14.3) | 1 (5.9) |
| Pneumonitis | 0 | 0 | 0 | 1 (14.3) | 1 (5.9) |
| Dry skin | 0 | 0 | 0 | 1 (14.3) | 1 (5.9) |
| Pruritus | 0 | 0 | 0 | 1 (14.3) | 1 (5.9) |
| Purpura | 0 | 0 | 0 | 1 (14.3) | 1 (5.9) |
| Toxic skin eruption | 0 | 0 | 0 | 1 (14.3) | 1 (5.9) |

^a^ Includes the AE terms neutropenia and neutrophil count decreased

^b^ Includes the AE terms leukopenia and white blood cell count decreased

^c^ Includes the AE terms thrombocytopenia and platelet count decreased

*AE* adverse event, *BID* twice daily, *INR* international normalized ratio, *QD* once daily

**ESM Table 3** Overall survival and progression-free survival according to the type of B-cell malignancy

|  | **Non-GCB DLBCL** | **DLBCL** | **MCL** | **FL** | **WM** | **CLL** |
| --- | --- | --- | --- | --- | --- | --- |
|  | ***N* = 4** | ***N* = 1** | ***N* = 4** | ***N* = 5** | ***N* = 2** | ***N* = 1** |
| ORR ^a^ |  |  |  |  |  |  |
| *n*/*N* | 3/4 | 1/1 | 4/4 | 2/5 | 2/2 | 1/1 |
| % (95% CI) | 75.0 (19.4–99.4) | 100.0 (2.5–100.0) | 100.0 (39.8–100.0) | 40.0 (5.3–85.3) | 100.0 (15.8–100.0) | 100.0 (2.5–100.0) |
| PFS, days, median (range) | 162 (5^+^–162^+^) | 341 (341–341) | Not reached (225–2018^+^) | 1030 (25–1030) | Not reached (841–1917^+^) | Not reached (1911^+^–1911^+^) |
| OS, days, median (range) | 239 (16–1392^+^) | 587 (587–587) | Not reached (443–2128^+^) | Not reached (214–1737^+^) | Not reached (1562–1917^+^) | Not reached (2076^+^–2076^+^) |

*CI* confidence interval, *CLL* chronic lymphocytic leukemia, *DLBCL* diffuse large B-cell lymphoma, *FL* follicular lymphoma, *MCL* mantle cell lymphoma, *non-GCB DLBCL* non-germinal center B-cell-like diffuse large B-cell lymphoma, *ORR* overall response rate, *OS* overall survival, *PFS* progression-free survival, *WM* Waldenström macroglobulinemia, + censored
